# Simulation studies of age-specific lifetime major depression prevalence

**DOI:** 10.1186/1471-244X-10-85

**Published:** 2010-10-20

**Authors:** Scott B Patten, Lee Gordon-Brown, Graham Meadows

**Affiliations:** 1 Department of Community Health Sciences & Department of Psychiatry, University of Calgary, 3330 Hospital Drive NW, Calgary, AB, T2N 4N1, Canada; 2 Econometrics & Business Statistics, Monash University, Melbourne, VIC, Australia; 3 Monash Univ, Sch Psychol Psychiat & Psychol Med, Melbourne, Vic 3004, Australia

## Abstract

**Background:**

The lifetime prevalence (LTP) of Major Depressive Disorder (MDD) is the proportion of a population having met criteria for MDD during their life up to the time of assessment. Expectation holds that LTP should increase with age, but this has not usually been observed. Instead, LTP typically increases in the teenage years and twenties, stabilizes in adulthood and then begins to decline in middle age. Proposed explanations for this pattern include: a cohort effect (increasing incidence in more recent birth cohorts), recall failure and/or differential mortality. Declining age-specific incidence may also play a role.

**Methods:**

We used a simulation model to explore patterns of incidence, recall and mortality in relation to the observed pattern of LTP. Lifetime prevalence estimates from the 2002 Canadian Community Health Survey, Mental Health and Wellbeing (CCHS 1.2) were used for model validation and calibration.

**Results:**

Incidence rates predicting realistic values for LTP in the 15-24 year age group (where mortality is unlikely to substantially influence prevalence) lead to excessive LTP later in life, given reasonable assumptions about mortality and recall failure. This suggests that (in the absence of cohort effects) incidence rates decline with age. Differential mortality may make a contribution to the prevalence pattern, but only in older age categories. Cohort effects can explain the observed pattern, but only if recent birth cohorts have a much higher (approximately 10-fold greater) risk and if incidence has increased with successive birth cohorts over the past 60-70 years.

**Conclusions:**

The pattern of lifetime prevalence observed in cross-sectional epidemiologic studies seems most plausibly explained by incidence that declines with age and where some respondents fail to recall past episodes. A cohort effect is not a necessary interpretation of the observed pattern of age-specific lifetime prevalence.

## Background

Psychiatric epidemiology is a relatively young discipline. A broad consensus on diagnostic definitions and associated approaches to measurement did not emerge until the 1980s with the publication of DSM-III [1]. In turn, DSM-III stimulated the development of fully structured diagnostic instruments, starting with the Diagnostic Interview Schedule (DIS) [2,3] and later the Composite International Diagnostic Interview (CIDI) [4,5]. The CIDI has continued to undergo modification and refinement [6], including adaptation for DSM-IV [7] diagnoses. A feature of both the DIS and the current version of the CIDI is a focus on lifetime prevalence (LTP): the proportion of a population that has met diagnostic criteria for a mental disorder during their life up to the time of assessment.

**Figure 1:**
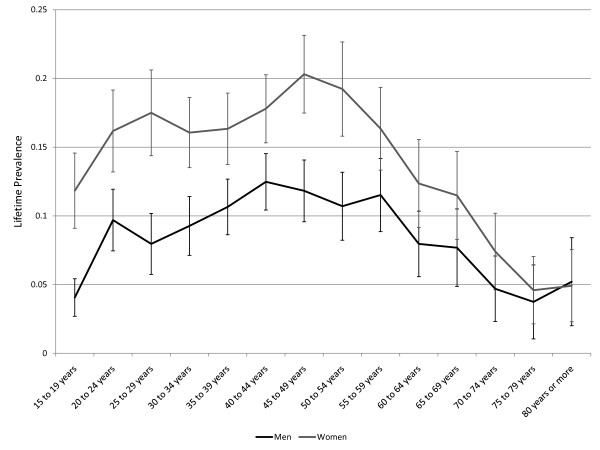
**Lifetime prevalence of major depression in the Canadian Community Health Survey 1.2, Mental Health and Wellbeing (error bars represent 95% confidence intervals)**.

Despite the emphasis on LTP during the past three decades, some basic questions about this parameter remain unanswered. One of the most problematic issues concerns the age-specific pattern of LTP for Major Depressive Disorder (MDD). MDD is irreversible by definition and expectation holds that LTP should increase with age. However, this pattern has not usually been observed. Instead, LTP has tended in most studies to increase during young adulthood, remain stable to early middle age, and to decline subsequently. Figure 1 presents the pattern of age-specific lifetime prevalence in men and women according to the Canadian Community Health Survey, Mental Health and Wellbeing (CCHS 1.2), which was conducted in 2002 [8].

There are several possible explanations for the observed pattern. A widely discussed possibility is that of a cohort effect: incidence may be increasing in more recent birth cohorts, leading to greater LTP in younger age groups. If this interpretation is correct, the decline in lifetime prevalence seen in older age groups is real, and future decades will be characterized by increasing prevalence in these groups as high-risk birth cohorts become older. The predicted secular trend is relevant to health service planning. Alternative explanations derive from the possibility of bias. Instruments that assess lifetime prevalence must rely on retrospective accounts of specific symptoms, their duration and severity. Existing instruments may not be able to accurately assess these aspects of respondents' personal histories. For example, Andrews et al. reported that the CIDI failed to detect prior episodes in up to 50% of cases 25 years after hospitalization for depression [9]. Failure to recall past symptoms may lead to LTP estimates that are biased downwards, a type of recall bias. An elevated rate of mortality in people with MDD could also theoretically lead to declining LTP in older age groups. The effect of MDD on mortality appears to be a modest one, however, with a relative risk of approximately 1.4 [10].

A series of "cradle to grave" cohort studies conducted in a succession of birth cohorts could unambiguously determine the origin of the observed LTP pattern. Such studies could theoretically avoid recall bias by avoiding the need for retrospective assessment. Such studies could also directly assess the impact of mortality on the age-specific estimates. However, such a series of cohort studies may not be practically feasible to conduct. Problems with the feasibility of "real world" studies provides a justification for the use of simulation techniques to examine these issues. The problem was first addressed using simulation in the 1990s by Giuffra and Risch [11] in a simulation study exploring the possible impact of recall bias on LTP. The modelling results reported by these authors confirmed that modest rates of "forgetting" (1% to 5% per year) could account for the emergence of cohort-like effects in Kaplan-Meier life tables. However, the Giuffra and Risch study was based on assumptions about incidence drawn from pre-DSM-III cohort studies. Some of these assumptions are inconsistent with more recent evidence. For example, 0.005 was used as an estimated annual risk in 16 to 20 year-olds, an estimate much lower than subsequent ones [12]. Also, these authors did not explore the possible impact of mortality in their simulations. A more recent simulation study based on 12-month prevalence data from Australia and the Netherlands found evidence of recall bias because projected LTP based on past month and past year data were much higher than reported LTP estimates [13]. This simulation study incorporated plausible values for mortality in its representation of the epidemiology.

The epidemiologic dynamics of MDD involve new onset cases (incidence) and removal of cases from the prevalence pool through mortality. Simulation involves developing a representation of this underlying "system." The use of simulation in this context is an appealing option because the underlying system is inherently simple. A widely used approach to simulation modeling, discrete event simulation, can represent a system of this sort by representing people as model entities. In discrete event simulation, entities can possess attributes (variables attached to those entities), allowing the depiction of different health states including age and prevalence.

In the current project, our goal was to (1) represent the epidemiology of MDD using a simulation model and (2) to explore the impact of changes to various input parameters on simulated patterns of age-specific LTP. While it is recognized that simulation cannot definitively disentangle the various potential explanations, the approach is useful because it can describe how various explanations may or may not fit together to produce observed patterns of LTP. As such, our goal was to identify whether the observed pattern of LTP can more or less plausibly result from various sets of assumptions concerning incidence, age effects and cohort effects.

## Methods

### Design of the Simulation Model

The model was an incidence-prevalence-mortality model in which age-specific LTP was represented as an outcome of age-specific incidence, age-specific mortality and a relative risk for mortality. A model of this type can support an assessment of age and cohort effects as incidence can be depicted as changing with age (age effect) or with time at birth (a cohort effect). A representation of excessive mortality risk was included in the model using a mortality ratio (MR): the ratio of death rates in those with MDD to those of the general population. The latter rates derived from vital statistics data. Discrete event simulation was used for the modeling, which was implemented in the software Arena, version 10 [14]. The simulation included a set of entities, representing people, each of whom were characterized by attributes reflecting their age, disease status and mortality status. We also incorporated a representation of recall bias into the model by allowing the lifetime prevalent cases to make a transition to a false negative state. False negative measurement status was also represented using an attribute. A more detailed description of the model is presented below.

a. Birth rate and age. Entities entered the simulation from a "create" module [14]. The time between entries was represented using an exponential distribution deriving from an arbitrary birth rate. This birth rate determines the size of the simulated population in its steady state condition but did not influence the simulated prevalence estimates. A simulated date of birth was recorded as an attribute for each entity using time on the simulation clock when the entity was created. Another attribute, the entity's age, was calculated as time on the simulation clock minus the entity's birth date.

b. Age-specific mortality. Age and sex-specific mortality statistics are available in Canada from the national statistical agency, Statistics Canada (http://www.statcan.gc.ca). An age of death was simulated for each entity by subjecting them to a mortality rate from the latest available national estimates for each year of their life [15]. Because mortality rates were available for five year age groups, entities were subjected to the relevant age and sex-specific mortality rates (using a series of "decide" modules). If they survived for five years, the entities moved to another age category where they were subjected to the next set of rates for the next five years and so on. Because a birth date was recorded as an attribute for each entity, the date of death could also be calculated and assigned (as an attribute) by adding the simulated duration of life to the birth date.

c. Age-specific incidence. After assignment of a date and age of death, the onset of disease was simulated in a similar manner. During each simulated year of life after age 15, each entity was exposed to a risk of new onset MDD. As MDD incidence in Canada may decline with age [16,17] the model was provided with flexibility to reflect this. The probability of incident MDD was depicted using two parameters, an initial risk (*C*) that would apply at the time of entry into the population at risk (which was assumed to be age 15 and the incidence was set at zero prior to this age) and another parameter (*r*) representing the extent to which the incidence declined as a function of age in years over the age of 15 (y), according to the following equation:

$$Incidence(y)=Ce^{-ry}$$

In order to represent distinct incidence in women and men, separate *C *and r parameters were used. With *r *set to zero, the incidence remains unchanged with age. An attribute was attached to each entity for the purpose of representing LTP. At birth the value of this attribute was set to zero. When an incidence of major depression occurred, this attribute value changed to one. If the simulated age of death occurred before the simulated age of onset of a disorder, the entity was considered to have remained free of MDD. It should be noted that the representation of incidence is monotonic, which is consistent with Canadian epidemiologic data. A more complex function would be required to represent complexities such as a potentially increased incidence in elderly age categories.

d. Age-specific lifetime prevalence. Entities in the model occupied a set of queues representing five year age groups (an exception being the first five years following birth, which was depicted using two separate queues since mortality is reported separately for the first year of life and for years 1-4 in Statistics Canada mortality tables). Arena can track the number of entities in a queue having a specified attribute. To represent age-specific LTP, the number of entities in an age group's queue possessing the attribute representing LTP was divided by the total number in the queue. These age-specific queues were made sex-specific, so that the model could simulate age and sex specific LTP (sex was also represented by an attribute attached to each entity at the time of birth). The model was run through a warm-up period in order to attain a steady state LTP. The simulation clock used days as a base-measure and the simulations were run for 100,000 days (approximately 274 years) in order to ensure that a steady state was reached. In reality, simulated LTP changed little when the simulations were run for long enough to replace the entire population. However, in simulations of cohort effects relevant to elderly age groups (e.g. cohorts born ninety years prior to the end of a simulation run) it was considered essential that the model be in steady state prior to these simulated births. For simplicity, all of the simulations used a 100,000 day simulation interval in order avoid a need to change the simulation interval for different simulations. An entity that survived a particular five year age interval moved to a queue representing the subsequent age group. Those that did not survive were removed from the model using a "dispose" module, leaving the queue at the simulated date of death.

e. Transition to false negative status. At the time of movement from one queue to the next, in other words at five year intervals, each entity was subjected to a probability of transition to false negative diagnostic status. False negative diagnostic status for an entity was represented using another attribute. The risk of transition to false negative status was a variable in the model, so that the effects of different false negative rates on "apparent" LTP (i.e. cases that would be detected despite false negative ratings using a diagnostic instrument) and actual LTP could be measured. Apparent and actual LPT were calculated using the same denominator (the number of entities in the queue), but with the false negative cases only being included in the actual LTP category.

f. Mortality ratio: When an entity developed MDD, their subsequent mortality was simulated using a model parameter that represented the elevated risk of mortality associated with MDD. This parameter, a mortality ratio (MR), was the ratio of age-specific death rates in lifetime depressed respondents divided by those in the general population. For example, if the MR was set at 2.0, then the mortality risk in any age group with MDD after the onset of the disorder would be twice that of the general population in that age group. After age-specific mortality rates for the LTP positive entities were calculated a date of mortality (and related attributes) was then re-simulated for these entities.

g. Cohort effects: Simulation effects were represented by altering model parameters for sets of entities created (i.e. "born") during specified time intervals as the simulation was running. For example, entities created 90 to 75 years prior to the end of a simulation comprised a birth cohort that was between 75 and 90 years old when the simulation run was over. Using this cohort as a baseline, relative risks were used to represent higher incidence in later birth cohorts.

**Figure 2:**
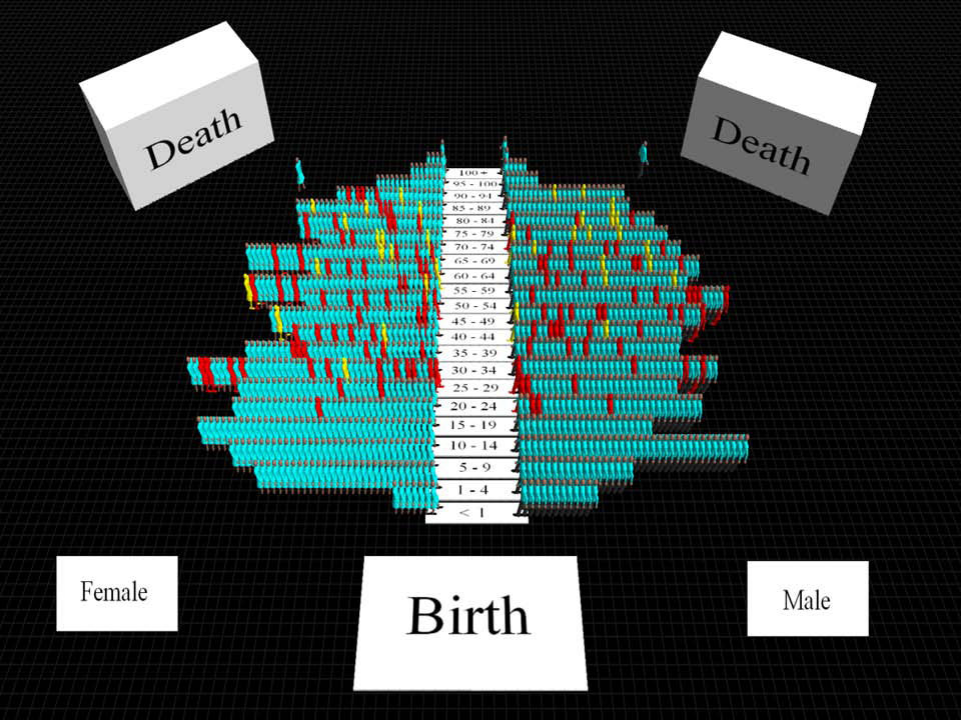
**Layout for animations of model simulations**.

An animation was developed for the model using the Arena 3D Player [14]. The various queues were depicted in the animation as a traditional "population pyramid" although, since the mortality rates in the model derived from a developed country, the shape was more cylindrical than pyramidal. Sex was depicted in the animation using different entity symbols for men and women, and LTP was depicted using a red colour for symbol representing the entity. False negative status was depicted using a yellow colour coding, see Figure 2.

### Validation of the Model

In order to be considered a valid representation of MDD epidemiology, it was necessary that the simulation model depict a pattern of LTP consistent with theoretical expectation. This included an expectation that: (1) LTP should increase with age (in any realistic scenario where *C *is greater than zero) so long as *r *was zero (incidence is constant with age), the relative risk of mortality is 1.0 (MDD does not influence mortality) and the false negative measurement risks are zero. (2) LTP should cease to increase at some age when *r *is large since age-specific incidence will eventually approach zero in this scenario, but so long as the relative risk of death and false negative measurement are unchanged the LTP should not decrease with age. (3) An increased relative risk for mortality or a sufficiently high false negative rate would both result in declining age-specific lifetime prevalence.

### Calibration of the Model

The Arena software includes an automated utility, called OptQuest, that can expedite the identification of sets of parameters achieving specified objectives. OptQuest runs a series of simulations while varying specified input parameters and seeking to find combinations of these input parameters that most closely reflect specified objectives. After validation, OptQuest was used to calibrate the simulation model under various sets of assumptions using the CCHS 1.2 estimates presented in Figure 1, above. A variable representing the sum of squares of simulated minus observed LTP (from the CCHS) summed separately for men and women across all of the age categories was used to identify values for the *C *parameter, *r*, the MR, and the false negative rate leading to simulated LTP pattern most closely resembling the observed pattern of age and sex-specific LTP. The simulated output representing apparent lifetime prevalence was used (ie. false negative diagnostic ratings were not counted in the denominator of the prevalence proportion) in these calibrations since the CCHS 1.2 data are subject to recall failure. In simulations exploring the ability of cohort effects to account for the observed pattern of LTP, the *r *parameters were set to zero, as was the probability of a false negative rating. This allowed OptQuest to identify the set of birth-cohort-specific relative risks that would best explain the observed pattern of LTP.

### Presentation of the Simulations

**Figure 3:**
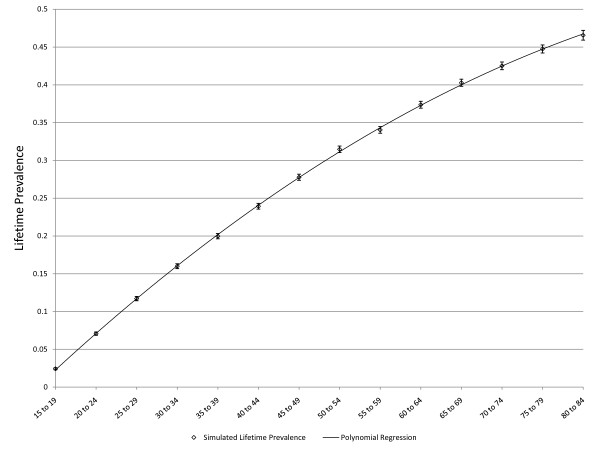
**Simulated age-specific LTP: constant incidence, no false negatives, no effect of depression on mortality**. C = 0.01, r = 0, false negative rate = 0, MR = 1, error bars represent 95% CIs.

**Figure 4:**
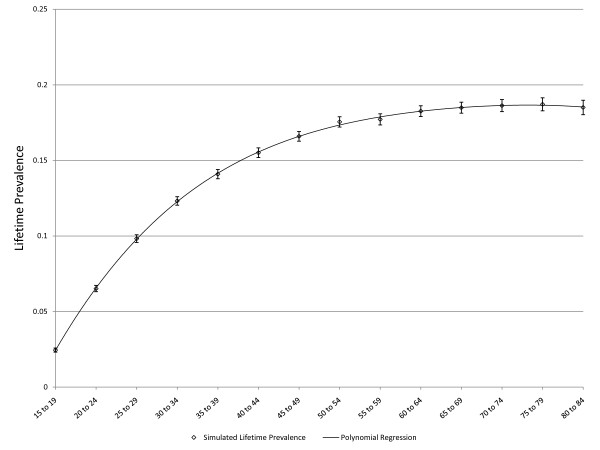
**Simulated age-specific LTP: declining incidence with age, no false negatives, no effect of depression on mortality**. C = 0.01, r = 0.05, false negative rate = 0, MR = 1, error bars represent 95% CIs

**Figure 5:**
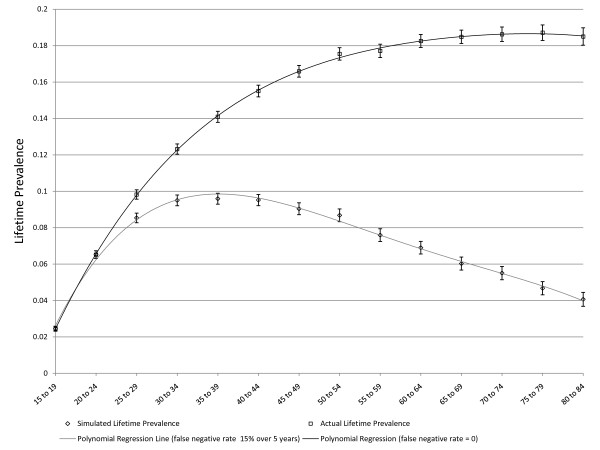
**Simulated age-specific LTP: declining incidence with age, 15% false negatives after 5 years, no effect of depression on mortality**. C = 0.01, r = 0.05, false negative rate 15% per 5 years, no effect of depression on mortality, error bars represent 95% CIs. One set of simulated values represents the actual lifetime prevalence, the other the apparent lifetime prevalence in which false negative results are not counted in the numerator of the prevalence proportion.

**Figure 6:**
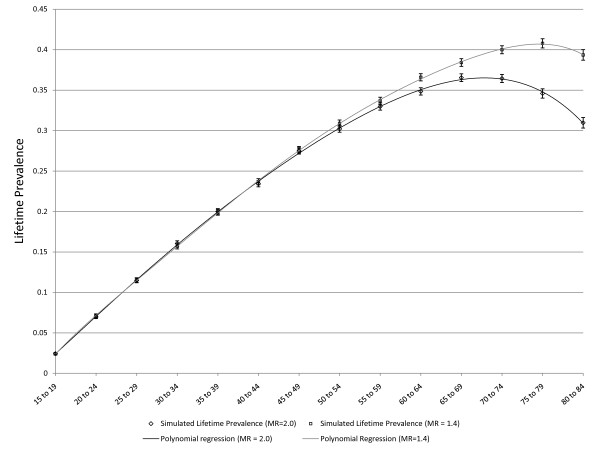
**Simulated age-specific LTP: effect of mortality with incidence that declines with age**. The dark line represents a strong effect of mortality (MR = 2.0) in the absence of false negative ratings and declining incidence: C = 0.01, r = 0, false negative rate = 0. The lighter line represents a more realistic effect of mortality (MR = 1.4) in the absence of false negative ratings and declining incidence: C = 0.01, r = 0, false negative rate = 0. The error bars represent 95% CIs.

**Figure 7:**
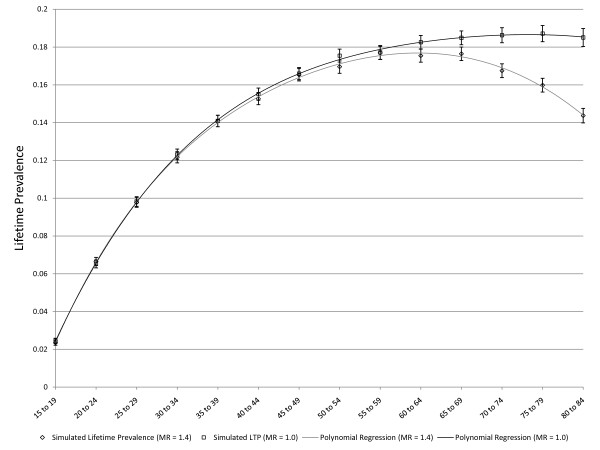
**Simulated age-specific LTP: effect of mortality with incidence that declines with age**. The dark line depicts constant incidence C = 0.01 that declines with age (r = 0.05) and there are no false negative ratings. This is the same simulation depicted in Figure 4 and is presented here for comparison to the lighter line, which represents a simulation based on the same assumptions except that MR is 1.4.

A simulation model consists of a series of statements about probabilities and is therefore akin to a set of population values, whereas the results of any particular simulation represent random variables arising from the model. As such, any particular simulation is subject to random error. For this reason, a set of n = 1000 simulations were run for most of the scenarios presented, and a 95% confidence interval based on the t-distribution is presented along with the simulation output for some of the simulations (Figure 3, Figure 4, Figure 5, Figure 6 and Figure 7, below), as recommended by Law [18]. An animation of the working simulation may be found here [19]. The animation runs at 864,000 times real time, so that ten days of simulation time pass by in 1 second of real time.

## Results

### Validation of the Model

As noted above, three scenarios in which the pattern of age-specific LTP could be predicted based on epidemiologic theory were explored for purposes of validation. Figure 3 presents simulated LTP with the C parameter for incidence set at 0.01 (1% per year), the MR set to one and the false negative risk set to zero. As expected, the lifetime prevalence increases with age. Figure 4 depicts simulated lifetime prevalence under the same set of assumptions but with the *r *parameter set to 0.05, depicting a 5% decline in incidence per year of age after age 15. As expected, the simulated lifetime prevalence fails to increase after several decades as the incidence becomes very small with increasing age but, consistent with expectation, LTP does not decrease. Figure 5 depicts the addition of a false negative risk of 15% per five year period (approximately 3% per year) in addition to the features of the second scenario (Figure 4). Including a false negative risk > 0 leads to deviation of actual from apparent LTP, both of which are depicted in the Figure. "Apparent" LTP does not include the false negative results in the numerator of the prevalence proportion, which produces an apparent decline in age-specific LTP. However, the actual LTP continues to increase and is identical to that depicted in Figure 4. While Figure 5 demonstrates that false negative diagnostic ratings can lead to an apparent decline in age-specific LTP when incidence declines with age, differential mortality is another possible explanation for this pattern. In the simulations depicted in Figure 6, the r parameter has been set to zero so that incidence does not decline with age and the rate of false negative ratings has also been set to zero. The Figure presents two simulations, one in which the MR is set to 1.4, consistent with existing literature, and one in which the MR is set to 2.0 (a value likely to be too high). Comparison of Figure 3 to Figure 6 confirms that differential mortality can affect age-specific LTP, but the effect tends to be evident only in elderly age groups. Combining the declining incidence depicted in Figure 4 with a MR of 1.4 leads to a lower LTP value and an earlier age for maximum LTP, see Figure 7, but the peak prevalence continues to occur at an older age group than has been reported by epidemiologic studies.

### Optimization

**Figure 8:**
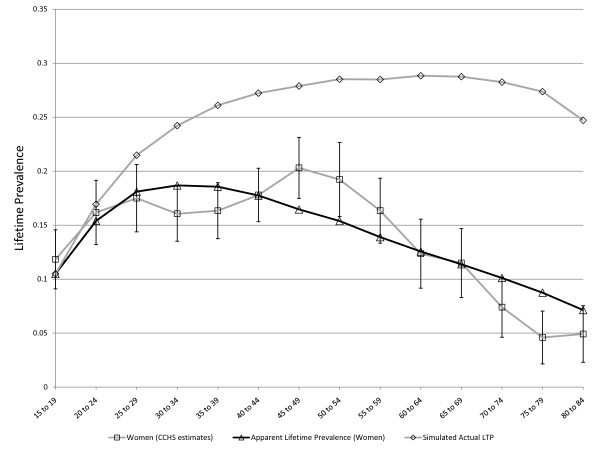
**Simulated age-specific LTP in women: model parameters optimized to CCHS 1.2 data**. C = 0.13, r = 0.08, MR = 1.2, FNR = 0.10. These parameter values derive from multiple simulations seeking to minimize the sum or squares of differences between simulated and observed age and sex-specific LTP estimates. The r parameter represents a decline in incidence with age > 15 (an age effect).

**Figure 9:**
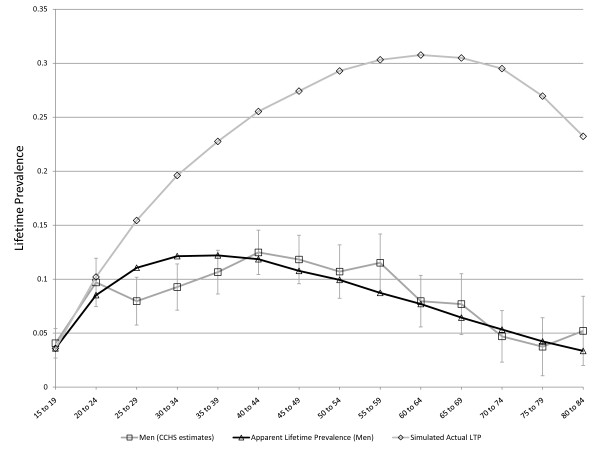
**Simulated age-specific LTP in men: model parameters optimized to CCHS 1.2 data**. C = 0.15, r = 0.03, MR = 1.7, FNR = 0.23. These parameter values derive from multiple simulations seeking to minimize the sum or squares of differences between simulated and observed age and sex-specific LTP estimates. The r parameter represents a decline in incidence with age > 15 (an age effect). The simulation includes an adjustment that places the LTP at 5% at the low end of the age range (age 15).

**Figure 10:**
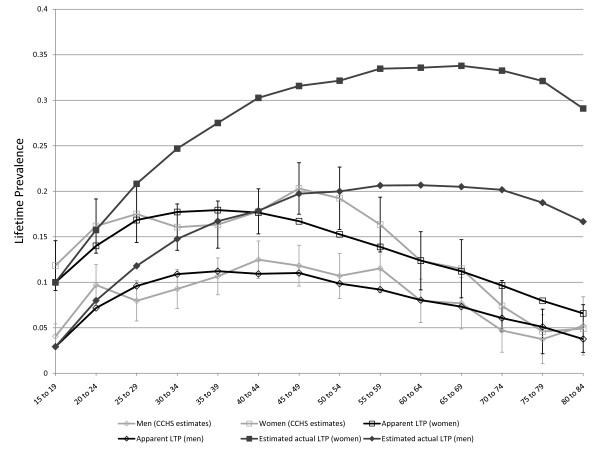
**Simulated and observed LTP in men and women and estimated actual LTP for men and women, with model constrained to a single value for the false negative rate**. The false negative rate is constrained to a single value, which was optimized at 0.14 per five year period, or approximately 3% per year.

As the results presented above are consistent with theory and support the validity of the model, OptQuest was used to calibrate the various parameters as described above. The overall MR was set at the realistic level of 1.4 [10] prior to the optimization. The high LTP in women in the youngest age group meant that a better fitting model could be achieved by allowing the baseline LTP in women at age 15 to be approximately 5% rather than starting at zero. OptQuest identified comparable *C *parameters for women than for men (0.020 compared to 0.015). In women, r was 0.084 compared to 0.034 in men. The sex-specific MR was 1.2 in women and 1.7 in men. Finally, the false negative rate was 0.10 in women (approximately 2% per year) and 0.23 (approximately 5% per year) in men. The optimization results suggest that the error rate in assessment of LTP may be higher in men than in women, consistent with previous reports indicating that the diagnosis of LTP is less reliable in men than women [20]. Figure 8 presents the observed and simulated values for LTP for women using these parameter values and Figure 9 represents the observed and simulated values for men. While the simulation model presented here was calibrated using a particular set of epidemiologic estimates (which were considered subject to false negative misclassification of diagnosis), it is also of some interest that the model output includes the actual lifetime prevalence. For this reason, Figure 8 and Figure 9 also show simulated age-specific LTP under the optimized values for the input parameters. The actual LTP proportions depicted are much greater than most published LTP estimates, but resemble estimates arising from previous simulation studies in women [13]. A prediction of the models depicted in Figure 8 and Figure 9 is that the actual lifetime prevalence in men and women may actually be comparable after about age 40, although apparent LTP continues to be higher in women. However, if the model is constrained to include a single false negative rate for men and women the optimized value is approximately 0.14 over 5 years (approximately 3% per year), and the simulated actual LTP peaks in the range of 30% for women and 20% for men, see Figure 10.

**Figure 11:**
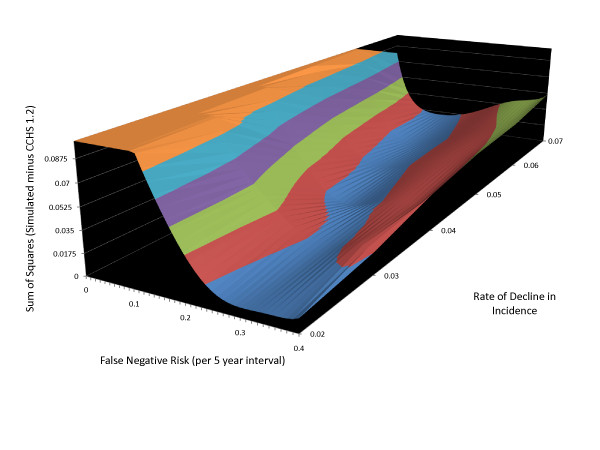
**Contour plot depicting model fit at various combinations of the false negative rate and r parameter**. The vertical axis is the sum of squared difference between observed age-specific LTP and simulated age-specific LTP. Lower elevation on the contour plot indicates a better concordance between observed and simulated age-specific LTP. The blue region at the lowest contour tracks diagonally across the plane at the base, indicating that higher r values provide a better fit when the false negative rate is low whereas lower r values provide a better fit when the false negative rate is higher.

Different combinations of model parameters can lead to similar patterns of simulated age-specific LTP. Figure 11 is a contour plot showing the sum of squares of differences between CCHS 1.2 and simulated LTP values at various combinations of values for these parameters and with the C parameter held constant at 0.012. The magnitude of the sum of squared differences is depicted on the vertical axis in relation to the false negative rate and rate of decline in incidence with increasing age on the horizontal axes. The lowest "altitude" on the vertical axes (depicted using the colour blue in the contour plot) represents a set of combinations of these two variables that minimize the sum of squares value. The plot shows a diagonal band in the blue contour, indicating that in circumstances of more rapidly declining incidence lower rates of false negative measurement are needed to accurately represent the CCHS 1.2 age-specific LTP estimates. With more slowly declining age-specific incidence, higher false negative measurement rates provide a better description of the observed pattern.

**Figure 12:**
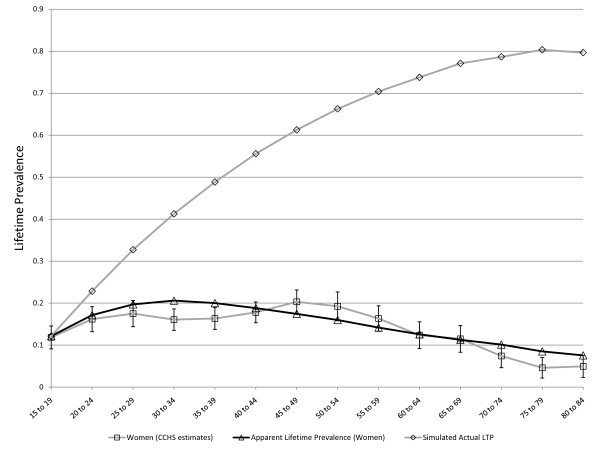
**Simulated age-specific LTP in women: model parameters optimized to CCHS 1.2 data under the assumption that incidence does not decline with age**. C = 0.14, r = 0.08, MR = 1.7, FNR = 0.33. These parameter values derive from multiple simulations seeking to minimize the sum or squares of differences between simulated and observed age and sex-specific LTP estimates.

**Figure 13:**
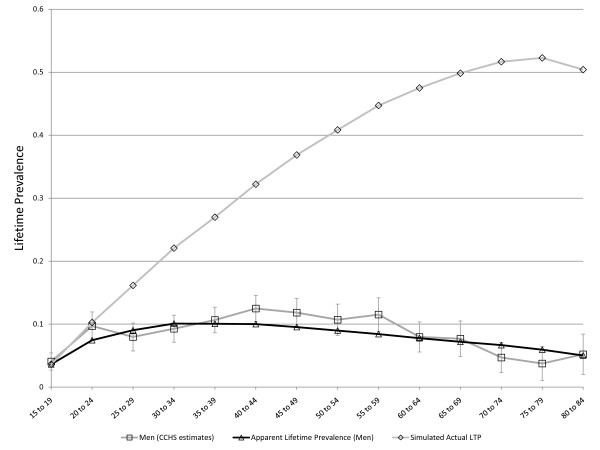
**Simulated age-specific LTP in men: model parameters optimized to CCHS 1.2 data under the assumption that incidence does not decline with age**. C = 0.14, r = 0.03, MR = 1.5, FNR = 0.40. These parameter values derive from multiple simulations seeking to minimize the sum or squares of differences between simulated and observed age and sex-specific LTP estimates. The r parameter represents a decline in incidence with age > 15 (an age effect).

It is theoretically possible that incidence does not decline with age, but that the false negative measurement rate is in itself sufficiently high to account for the observed pattern. In order to explore this possibility, additional optimizations were used to identify estimates for the input parameters, but setting the r parameter to zero. This did not result in large changes to the C parameter or to the MR. In men, the optimized parameters were found to be 0.014 and 1.52 respectively and in women they were 0.028 and 1.68 respectively. The optimized false negative rate was much higher than that identified in optimizations allowing incidence to diminish with age: in men this was found to be 41% per five years, or approximately 10% per year, whereas in women it was 33% per five years, or approximately 8% per year. These false negative rates seem excessively high and would need to be associated with very high actual LTP values in order to produce the observed pattern. Figure 12 and Figure 13 show the optimized simulations under this set of assumptions for: women (Figure 12) and men (Figure 13).

### Cohort Effects

The pattern of declining incidence with age employed in the simulations reported above cannot be distinguished from increasing incidence in more recent birth cohorts based on the degree of fit with CCHS 1.2 data because each annual birth cohort is represented by a single age category in the cross-sectional CCHS 1.2 data. For this reason, the simulations reported above essentially assume that there are no cohort effects. A series of simulations reported in the following section of this paper assume constant age-specific incidence in order to explore the impact of cohort effects.

As described above, OptQuest was used to identify plausible values for relative risks associated with 10-year birth cohort categories. The 75 years or older category was treated as a baseline group for each relative risk value, such that the relative risks represent the incidence affecting more recent birth cohorts relative to those of the 75+ age group. In these simulations, the r parameter and the false negative rate were both set to zero, so that changes in age-specific lifetime prevalence could be attributable only to birth cohort effects. The relative risk estimates identified by OptQuest are presented in Table 1.

**Table 1:** Relative risks by birth cohort identified by OptQuest based on CCHS 1.2 data

Birth cohort	Age in 2002*	Relative Risk
Birth cohort after 1977	< 25	11.2
Birth cohort 1967 to 1976	25 to 34.9	6.4
Birth cohort 1957 to 1966	35 to 44.9	4.9
Birth cohort 1947 to 1956	45 to 54.9	5.3
Birth cohort 1937 to 1946	55 to 64.9	2.8
Birth cohort 1927 to 1936	65 to 74.9	1.7
Birth cohort before 1926	75 and older	baseline

* year of the CCHS 1.2 survey.

**Figure 14:**
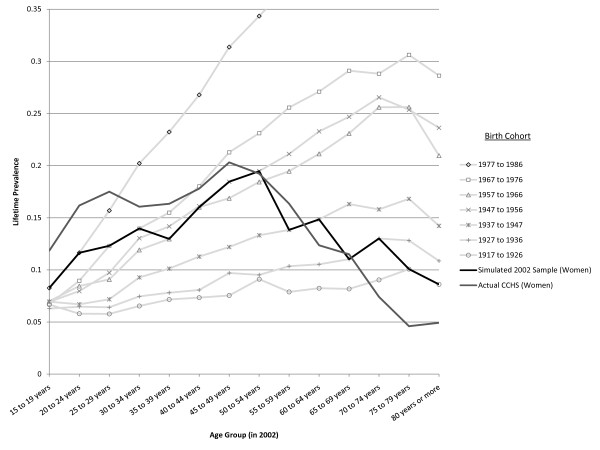
**Simulated age- and sex-specific LTP, by birth cohort in women**. The light lines represent the projected age-specific LTP pattern experienced by the birth cohorts assuming no change in incidence with age (r = 0) and no false negative ratings. The MR is set at 1.4 in these simulations.

**Figure 15:**
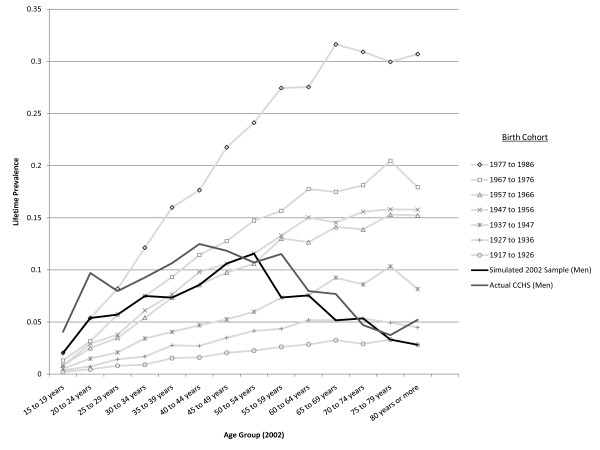
**Simulated age- and sex-specific LTP, by birth cohort in men**. The light lines represent the projected age-specific LTP pattern experienced by the birth cohorts assuming no change in incidence with age (r = 0) and no false negative ratings. The MR is set at 1.4 in these simulations.

The optimized simulation for cohort effects is presented in Figure 14 (women) and Figure 15 (men) showing the simulated experience of each birth cohort. The black line super-imposed on the plot is the LTP value for each age cohort at a specific year (taken here to represent the 2002 year of the CCHS 1.2 survey). For comparison, a grey line showing the actual LTP estimates (from the CCHS 1.2) is also presented on the Figures. Because the representation of birth cohorts was made using 10-year intervals and the age-specific LTP is simulated in 5-year intervals, the dark line (representing age-specific cross-sectional data), coincides with the experience of a particular birth cohort at two data points. The cohort effect does produce the pattern of LTP seen in the epidemiologic data. The dramatic cohort effects that must underpin this pattern are depicted by the light grey lines showing the pattern of age-specific LTP identified by the birth cohort relative risks in Table 1.

Table 1 indicates that a cohort effect is consistent with the observed age-specific pattern of LTP under certain circumstances. A cohort effect impacting only young people is not consistent with the observed pattern, instead, there must be a progressive increase in the relative risk associated with birth cohorts more recent than 1926. Also, the extent of the cohort effect must be very strong if it is to explain the pattern. The relative risk in the youngest birth cohort, corresponding to those aged 15-25 in the 2002 survey must have more than a 10-fold greater increase in incidence relative to the baseline group. This means that if the pattern of LTP is due exclusively to a cohort effect, dramatic increases in prevalence will be seen in elderly age groups in upcoming decades. On Figure 14 and Figure 15 moving from the dark line up to the next light grey line shows the model's projection of LPT after 10 years, moving up two of the lines shows the projection for twenty years into the future and so on. Women have a peak prevalence of slightly less than 20% in the 45-55 year old age group, which is projected to increase to 30-35% over the next 20 years under the assumption that the age-specific LTP pattern is due to cohort effects. An animation depicting the predicted age-specific LTP pattern over 60 years given the cohort effect assumption may be found here [21].

## Discussion

The World Mental Health Surveys Initiative has recently reported results for the first 17 of the (>30) countries involved in the initiative [22]. While reported lifetime prevalence varied considerably across countries, most countries reported an age-specific pattern of LTP similar to that seen in the CCHS 1.2, an increase in lifetime prevalence among the youngest age categories followed by a decline in older age groups. Cohort effects were the preferred interpretation of this pattern by most authors [22]. The simulation models presented here demonstrate that the pattern of LTP can be explained by a combination of declining incidence with age and a modest false negative rate in diagnostic assessment. While these studies cannot confirm or deny the possibility of cohort effects, they do illustrate that the cohort interpretation is not necessary to explain the observed pattern. Furthermore, an explanation based on a cohort effect must involve two elements that have not previously been identified: (1) the cohort effects must have had their onset not with recent birth cohorts. Instead, the increase in incidence by birth cohort must have been going on since the time of birth of the eldest members of the current population, (2) the cohort effect must be very large in magnitude, with younger birth cohorts being subject to incidence rates approximately 10 times greater than those of earlier birth cohorts.

These results are of importance to health policy, since a cohort effect on MDD prevalence would be of importance for public health. The cohort interpretation implies that recent birth cohorts will cause a dramatic increasing prevalence of MDD in successively older age groups as these birth cohorts age during upcoming decades. On the other hand, the results of this simulation study, and others [11,13] indicate that the observed pattern may reflect several factors, including measurement artefact.

The idea that the incidence of MDD declines with age was built into the simulation model by inclusion of a parameter reflecting a rate of decline in incidence with increasing age. In the model, incidence that does not decline with age is a special instance in which the *r *parameter is equal to zero. The decision to build the possibility of declining incidence into the model was based on findings from a Canadian longitudinal study [16,17]. The same pattern was also observed, however, in the Baltimore follow-up component of the US Epidemiologic Catchment Area studies [23] in men and to a lesser extent in women. In that study the peak incidence for women was in the 30-44 age group. Similarly, data from the Netherlands Mental Health Survey and Incidence Study (NEMESIS) indicated peak incidence in men in the 25-34 age group and the 35-44 age group in women [24]. However, each of these studies used LTP as an exclusion criterion in their assessment of eligibility for first incidence during baseline assessments. As a corollary of the observation that recall failure probably occurs with this type of measure, these ages of onset are almost certainly biased upwards by misclassification of recurrence as first incidence. These ideas are also consistent with studies of MDD prevalence in adolescents and young adults that have usually reported LTP estimates similar to those reported for general population adult samples [25-27].

The possibility of false negative diagnostic assessment was built into the simulation model. In the calibration process, a value for this risk was selected based on the pattern of LTP observed in the CCHS 1.2, and with simulations incorporating what was considered to be a reasonable level differential mortality. The values arrived at appear realistic based on the published literature. The Andrews et al. study of recall of earlier depressive episodes [9] took place over 25 years and found that approximately half of the participants did not meet CIDI criteria. Using the equation: cumulative risk over 25 years = 1-(1-*annualrisk*)^25 ^leads to a projection of 53% when the annual risk is 3% which falls between the estimates for men and women arising from this study.

Supporting evidence has also recently been reported by Moffitt et al. using data from the Dunedin birth cohort. These authors reported that members of this cohort, followed prospectively to age 32, had an estimated lifetime prevalence double that of retrospectively ascertained lifetime prevalence (41.4% versus 18.5%) [28]. This observation is consistent with the idea that false negative measurement errors bias LTP estimates from cross-sectional studies downwards.

What role can simulation play in understanding major depression epidemiology? Simulation studies cannot definitively determine the extent to which cohort effects, mortality effects or measurement bias determine the observed pattern of age-specific LTP. However, by allowing "what if" scenarios to be examined under various sets of assumptions they can support interpretation of the available estimates. The simulations presented here indicate that differential mortality probably makes only a minor contribution to the observed pattern. Even an implausibly large extent of differential mortality cannot account for the decline in age-specific LTP that starts in middle age. If incidence declines with age it follows that measurement bias, at a level similar to that reported by previous studies, can account for the observed pattern of age-specific LTP without resorting to cohort effects as an explanation. Cohort effects may contribute to the observed pattern, but the magnitude of difference in incidence in different birth cohorts would appear to make such effects unlikely candidates as the sole factor for diminishing age-specific LTP.

## Conclusions

The main implication of these results is that a cohort effect is not the only plausible explanation for the observed pattern of age-specific lifetime prevalence. Mental health surveys that interpret their results as providing evidence of cohort effects create an expectation that prevalence will increase dramatically in upcoming decades, particularly in older age groups. The results presented here indicate that this interpretation may be premature and that the projected increase may not occur.

## Competing interests

The authors declare that they have no competing interests.

## Authors' contributions

All three authors contributed to the conception of the study and each has read the final draft of the paper. SP developed the simulation model in consultation with LGB and SP ran the simulations presented in the paper. SP also wrote the initial draft of the paper. Both LGB and GM provided feedback on drafts of the manuscript and the simulations.

## Pre-publication history

The pre-publication history for this paper can be accessed here:

http://www.biomedcentral.com/1471-244X/10/85/prepub
